# Direct bilirubin levels are prognostic in non-small cell lung cancer

**DOI:** 10.18632/oncotarget.23184

**Published:** 2017-12-12

**Authors:** Ying-Jian Song, Xin-Huai Gao, Yong-Qing Hong, Li-Xin Wang

**Affiliations:** ^1^ Department of Respiratory Medicine, Huai’an First People’s Hospital, Nanjing Medical University, Huai’an 223300, Jiangsu, China

**Keywords:** non-small cell lung cancer, bilirubin, chemotherapy, outcome, nomogram

## Abstract

We investigated the prognostic value of serum bilirubin levels in stage I–II non-small cell lung cancer (NSCLC) patients and evaluated the relationship between bilirubin levels and response to first-line platinum-based chemotherapy. We divided 634 NSCLC patients from a single hospital in China into retrospective training (*n* = 307) and prospective validation (*n* = 327) cohorts. X-tile was used to identify the optimal serum bilirubin cutoff value for sorting retrospective cohort patients into low and high overall survival (OS) groups. TNM stage and serum bilirubin levels were associated with OS on univariate analysis. Direct bilirubin (DBIL) levels were correlated with tumor progression and response to first-line platinum-based chemotherapy, and were associated with OS after adjusting for TNM stage. Our findings indicate a DBIL-based prognostic nomogram is more accurate than the TNM staging system in predicting clinical outcomes, and that the DBIL level is an independent predictor of OS in NSCLC. Thus, an index that combines DBIL with TNM stage may better predict patient outcomes than TNM stage alone.

## INTRODUCTION

Lung cancer is the leading cause of cancer death worldwide [1]. Non-small cell lung cancer (NSCLC) accounts for approximately 85% of lung cancer cases in the U.S. [2]. Predicting NSCLC patient outcomes is challenging due to differences in genetic, clinical, and environmental factors [3]. Many NSCLC patients are diagnosed at a late stage and experience relapse and disease progression following surgical resection [4].

Prognostic predictions in NSCLC are based on the tumor-node-metastasis (TNM) staging system. However, this system cannot account for several factors that impact patient outcomes including clinical characteristics, lab results, and treatment regimens. Prediction tools such as nomograms have been developed to predict prognosis in patients with various cancers. However, they are not routinely used in NSCLC.

Serum bilirubin is an end product of heme metabolism. It has been shown to have potent antioxidant and antitumor effects in lung cancer [5, 6]. Studies of the relationships between circulating antioxidant levels and cancer risk have yielded conflicting results. Baseline serum bilirubin levels were inversely correlated with cancer mortality in a Belgian population [7]. Additionally, two prospective studies reported an inverse association between serum bilirubin levels and lung cancer risk [8, 9].

In this study, we explored the relationship between serum bilirubin levels and clinical outcomes following surgical resection in early-stage (I–II) NSCLC patients. We also evaluated the association between serum bilirubin levels and response to first-line platinum-based chemotherapy. Finally, we developed a serum bilirubin-based nomogram that could more accurately predict 5-year clinical outcomes in these patients than TNM stage alone.

## RESULTS

### Patient characteristics

NSCLC patients from a single hospital in China were divided into retrospective training (*n* = 307) and prospective validation (*n* = 327) cohorts. The baseline characteristics of all patients are shown in Table 1. The training cohort consisted of 102 men and 205 women. Of these patients, 193 (62.87%) were histologically diagnosed with adenocarcinoma and 187 (60.91%) were treated with first-line chemotherapy. The mean patient age was 65 ± 12 years. A total of 146 (47.56%) patients in the training cohort died during follow-up. The TNM stage distribution varied widely in the validation cohort: 93 patients (28.44%) had early-stage NSCLC and 131 (40.06%) had node-negative disease. A total of 143 (43.73%) patients in the validation cohort died during follow-up (median follow-up period, 1,085 days).

**Table 1:** Clinical characteristics for training and validation cohort patients

Baseline characteristics	Training cohort *N* = 307	Validation cohort *N* = 327	*P* for value
Age (years)	64 (57–72)	65 (57–74)	0.546
Sex (male)	102 (33.22)	125 (38.23)	0.179
Smoking	58 (18.89)	74 (22.63)	0.169
ECOG PS			
0–1	247 (80.46)	266 (81.35)	0.791
2–3	60 (19.54)	61 (18.65)	
Histological type			
Adenocarcinoma	193 (62.87)	213 (65.14)	0.516
Non-adenocarcinoma	114 (37.13)	114 (34.86)	
TNM stage (I/II/III)	99/106/102	93/99/135	0.111
pT stage (1/2/3/4)	60/44/181/22	72/59/160/36	0.062
pN stage (0/1/2/3)	128/93/54/32	131/113/57/26	0.562
chemotherapy	187 (60.91)	175 (53.52)	0.067
CEA (ng/mL)			
<5	200 (65.15)	194 (59.33)	0.146
≥5	107 (34.85)	133 (40.67)	
TBIL (μmol/L)	9.7 (6.8–14.4)	10.0 (6.8–15.2)	0.114
DBIL (μmol/L)	3.1 (2.1–4.5)	3.4 (2.4–5.2)	0.356
IBIL (μmol/L)	6.6 (4.0–9.1)	6.4 (4.2–9.8)	0.442

ECOG PS, Eastern Cooperative Oncology Group performance status; TBIL, total bilirubin; DBIL, direct bilirubin; IBIL, indirect bilirubin.

All data were analyzed using χ^2^ test or Mann-Whitney *U* test.

Values are expressed as medians (interquartile range) or frequencies and percentages.

### Correlation between serum bilirubin levels and the baseline characteristics of NSCLC patients

**Figure 1:**
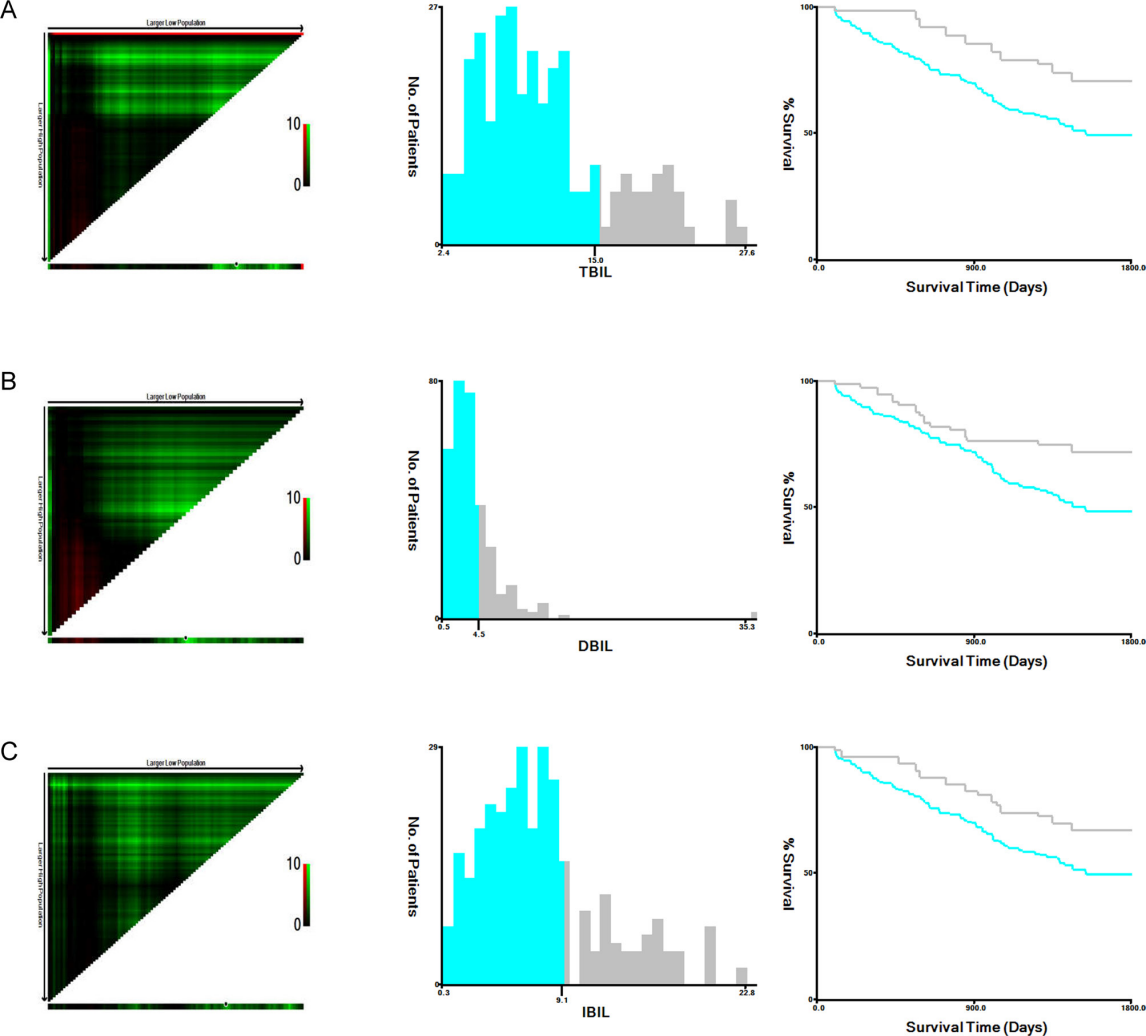
Analysis of TBIL **(A)**, DBIL **(B)**, and IBIL **(C)** levels in the training cohort using X-tile. X-tile plots (left panels); optimal cutoffs (black circles, middle panels); Kaplan-Meier plots (right panels).

The baseline characteristics of the patients in the training and validation cohorts were similar (*P* > 0.05) (Table 1). The X-tile software was used to identify the optimal serum bilirubin cutoff value for sorting patients in the training cohort into low and high bilirubin groups: 15 μmol/L for total bilirubin (TBIL), 4.5 μmol/L for direct bilirubin (DBIL), and 9.1 μmol/L for indirect bilirubin (IBIL) (Figure 1). Pretreatment DBIL levels were associated with TNM stage, pT stage, pN stage, and chemotherapy in both the training and validation cohorts (Table 3). However, no correlation between pretreatment TBIL and IBIL levels and patient baseline characteristics was observed in either cohort (Tables 2 and 4).

**Table 2:** Distribution of baseline characteristics stratified by pretreatment TBIL

Baseline characteristics	Training cohort			Validation cohort		
	Low TBIL	High TBIL	*P*	Low TBIL	High TBIL	*P*
Age (years)	63 (56–72)	64 (55–73)	0.324	65 (57–75)	64 (56–74)	0.096
Sex (male)	81 (34.47)	21 (29.17)	0.403	85 (35.12)	40 (47.06)	0.051
Smoking	42 (17.87)	16 (22.22)	0.409	52 (21.49)	22 (25.88)	0.405
ECOG PS						
0–1	185 (78.72)	62 (86.11)	0.167	201 (83.06)	65 (76.47)	0.180
2–3	50 (21.28)	10 (13.89)		41 (16.94)	20 (23.53)	
Histological type						
Adenocarcinoma	152 (64.68)	41 (56.94)	0.235	162 (66.94)	51 (60.00)	0.248
Non-adenocarcinoma	83 (35.32)	31 (43.06)		80 (33.06)	34 (40.00)	
TNM stage (I/II/III)	80/79/76	19/27/26	0.478	66/73/103	27/26/32	0.692
pT stage (1/2/3/4)	43/34/142/16	17/10/39/6	0.716	58/41/119/24	14/18/41/12	0.355
pN stage (0/1/2/3)	96/72/45/22	32/21/9/10	0.444	95/89/41/17	36/24/16/9	0.456
chemotherapy	149 (63.40)	38 (52.78)	0.106	123 (50.83)	52 (61.18)	0.100
CEA (ng/mL)						
<5	158 (67.23)	42 (58.33)	0.166	138 (57.02)	56 (65.88)	0.153
≥5	77 (32.77)	30 (41.67)		104 (42.98)	29 (34.12)	

ECOG PS, Eastern Cooperative Oncology Group performance status; TBIL, total bilirubin.

All data were analyzed using χ^2^ test or Mann-Whitney *U* test.

Values are expressed as medians (interquartile range) or frequencies and percentages.

**Table 3:** Distribution of baseline characteristics stratified by pretreatment DBIL

Baseline characteristics	Training cohort			Validation cohort		
	Low DBIL	High DBIL	*P*	Low DBIL	High DBIL	*P*
Age (years)	64 (56–73)	64 (55–73)	0.196	64 (56–75)	65 (56–74)	0.259
Sex (male)	75 (33.33)	27 (32.93)	0.947	68 (31.34)	34 (30.91)	0.937
Smoking	38 (16.89)	20 (24.39)	0.137	45 (20.74)	29 (26.36)	0.251
ECOG PS						
0–1	180 (80.00)	67 (81.71)	0.739	175 (80.65)	91 (82.73)	0.648
2–3	45 (20.00)	15 (18.29)		42 (19.35)	19 (17.27)	
Histological type						
Adenocarcinoma	142 (63.11)	51 (62.20)	0.883	140 (64.52)	73 (66.36)	0.740
Non-adenocarcinoma	83 (36.89)	31 (37.80)		77 (35.48)	37 (33.64)	
TNM stage (I/II/III)	61/82/82	38/24/20	0.005	54/76/97	39/23/38	0.014
pT stage (1/2/3/4)	32/28/171/14	28/16/10/8	<0.001	42/25/128/22	30/34/32/14	<0.001
pN stage (0/1/2/3)	80/73/42/15	48/20/12/17	0.001	78/92/28/19	53/21/29/7	<0.001
chemotherapy	151 (67.11)	36 (43.90)	<0.001	148 (68.20)	27 (24.55)	<0.001
CEA (ng/mL)						
<5	140 (62.22)	60 (73.17)	0.075	135 (62.21)	59 (53.64)	0.136
≥5	85 (37.78)	22 (26.83)		82 (37.79)	51 (46.36)	

ECOG PS, Eastern Cooperative Oncology Group performance status; DBIL, direct bilirubin.

All data were analyzed using χ^2^ test or Mann-Whitney *U* test.

Values are expressed as medians (interquartile range) or frequencies and percentages.

**Table 4:** Distribution of baseline characteristics stratified by pretreatment IBIL

Baseline characteristics	Training cohort			Validation cohort		
	Low IBIL	High IBIL	*P*	Low IBIL	High IBIL	*P*
Age (years)	64 (56–73)	64 (54–73)	0.180	64 (56–75)	64 (56–73)	0.098
Sex (male)	75 (33.19)	27 (33.33)	0.981	95 (41.48)	30 (30.61)	0.064
Smoking	39 (17.26)	19 (23.46)	0.221	49 (21.40)	25 (25.51)	0.415
ECOG PS						
0–1	142 (62.83)	55 (67.90)	0.414	180 (78.60)	86 (87.76)	0.052
2–3	84 (37.17)	26 (32.10)		49 (21.40)	12 (12.24)	
Histological type						
Adenocarcinoma	145 (64.16)	48 (59.26)	0.434	151 (65.94)	62 (63.27)	0.642
Non-adenocarcinoma	81 (35.84)	33 (40.74)		78 (34.06)	36 (36.73)	
TNM stage (I/II/III)	78/78/70	21/28/32	0.263	67/70/92	26/29/43	0.807
pT stage (1/2/3/4)	50/32/131/13	10/12/50/9	0.141	52/32/116/29	20/27/44/7	0.023
pN stage (0/1/2/3)	98/73/32/23	30/20/22/9	0.058	84/84/39/22	47/29/18/4	0.117
chemotherapy	144 (63.72)	43 (53.09)	0.093	149 (65.07)	26 (26.53)	<0.001
CEA (ng/mL)						
<5	148 (65.49)	52 (64.20)	0.835	140 (61.14)	54 (55.10)	0.309
≥5	78 (34.51)	29 (35.80)		89 (38.86)	44 (44.90)	

ECOG PS, Eastern Cooperative Oncology Group performance status; IBIL, indirect bilirubin.

All data were analyzed using χ^2^ test or Mann-Whitney *U* test.

Values are expressed as medians (interquartile range) or frequencies and percentages.

### Association between DBIL levels and response to first-line platinum-based combination chemotherapy

We next investigated whether pretreatment DBIL levels were associated with the response to first-line platinum-based combination chemotherapy (Table 5). Complete response was obtained after two cycles of chemotherapy in 11 (7.43%) patients in the low DBIL group compared to three (11.11%) in the high DBIL group. Partial response was also more common in the high DBIL group (51.85% vs. 29.05%). There were 94 patients (63.51%) in the low DBIL group who were non-responders compared to 10 (37.03%) in the high DBIL group (*P* = 0.010).

**Table 5:** Treatment response in 327 NSCLC patients treated with first-line platinum-based chemotherapy

Treatment response	DBIL		*P*
	Low DBIL (*n* = 148)	High DBIL (*n* = 27)	
CR	11 (7.43)	3 (11.11)	0.045
PR	43 (29.05)	14 (51.85)	
SD	59 (39.86)	4 (14.81)	
PD	35 (23.65)	6 (22.22)	
Responder (CR and PR)	54 (36.49)	17 (62.96)	0.010
Non-responder (SD and PD)	94 (63.51)	10 (37.03)	

CR complete response, PR partial response, SD stable disease, PD progressive disease.

All data were analyzed using χ^2^ test.

### Prognostic significance of serum bilirubin levels

**Figure 2:**
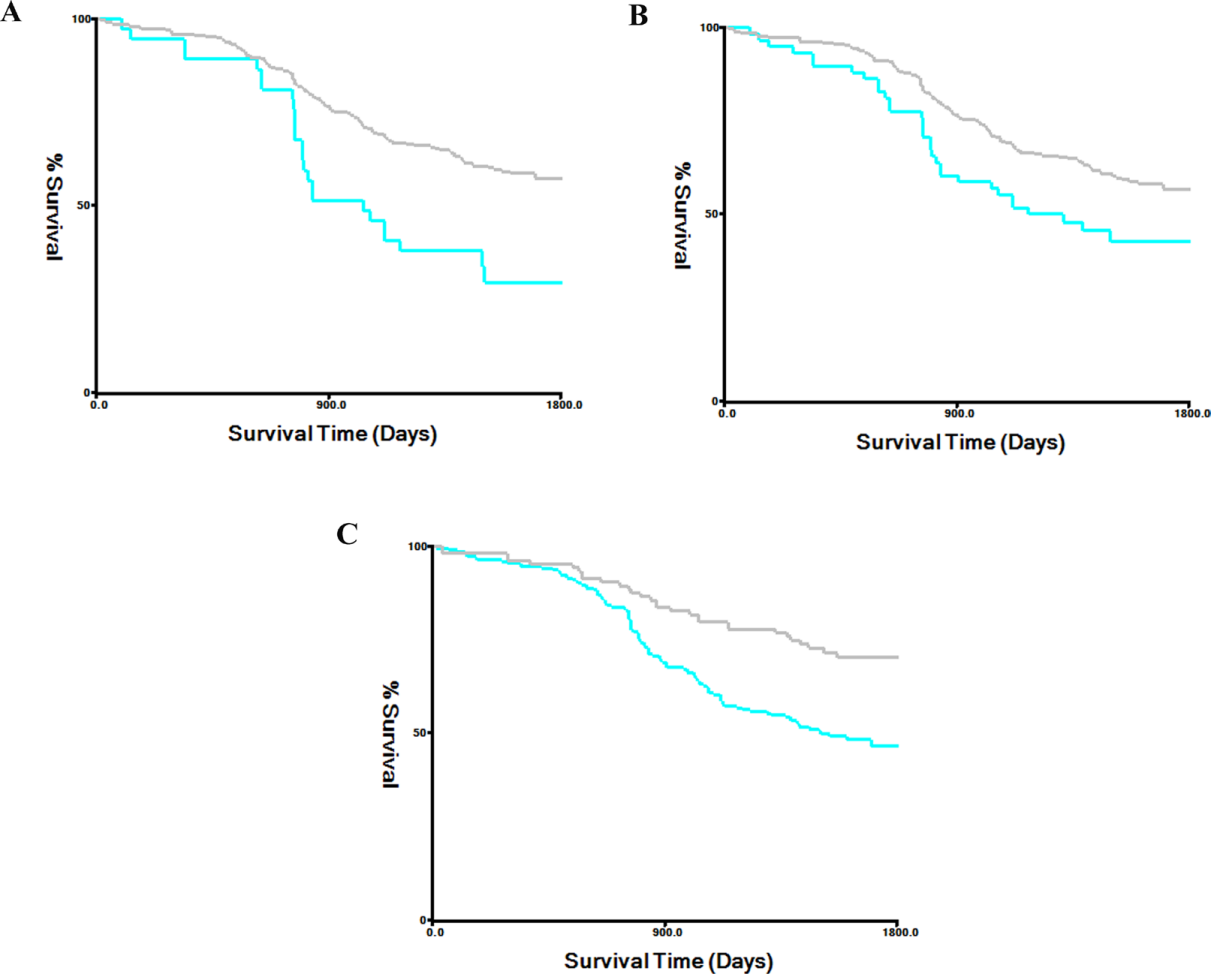
Kaplan-Meier survival curves stratified by serum bilirubin level in the validation cohort

Univariate log-rank tests for clinical prognostic factors in the training and validation cohorts are shown in Table 6. The prognostic factors that were significant on univariate analysis were TNM stage, pT stage, pN stage, chemotherapy, TBIL, DBIL, and IBIL. Kaplan-Meier survival curves stratified by serum bilirubin level are shown in Figures 1 and 2 (all *P*-values > 0.05). Multivariate Cox regression analysis confirmed that TNM stage, pT stage, pN stage, and DBIL were independent prognostic indicators for OS in both the training and validation cohorts.

**Table 6:** Univariate and multivariate analyses of prognostic value of serum bilirubin

Factors	Training cohort			Validation cohort		
	Univariate analysis	Multivariate analysis		Univariate analysis	Multivariate analysis	
	*P*	HR (95%CI)	*P*	*P*	HR (95%CI)	*P*
Age (years)	0.085			0.234		
Sex (male)	0.124			0.162		
Smoking	0.541			0.324		
ECOG PS (2–3)	0.594			0.160		
Histological type	0.220			0.108		
TNM stage	<0.001			<0.001		
I		Reference			Reference	
II		1.24 (0.96–3.25)	0.057		1.30 (1.11–2.64)	0.020
III		2.59 (1.20–4.98)	<0.001		2.42 (1.14–3.58)	<0.001
pT stage	<0.001			<0.001		
T1–2		Reference			Reference	
T3–4		2.45 (1.22–5.19)	<0.001		1.99 (1.12–3.20)	0.002
pN stage	<0.001			<0.001		
T0–1		Reference			Reference	
T2–3		1.86 (1.08–2.80)	0.005		2.00 (1.20–3.52)	<0.001
chemotherapy	0.042	1.14 (0.95–1.86)	0.089	0.030	1.23 (0.82–1.64)	0.124
CEA (abnormal)	0.385			0.110		
TBIL (≥15 μmol/L)	0.005	0.98 (0.76–1.24)	0.582	0.012	1.01 (0.65–1.09)	0.101
DBIL (≥4.5 μmol/L)	<0.001	0.64 (0.52–0.91)	0.004	0.004	0.74 (0.61–0.88)	<0.001
IBIL (≥9.1 μmol/L)	0.032	1.00 (0.80–1.35)	0.532	0.001	0.94 (0.56–1.02)	0.098

ECOG PS, Eastern Cooperative Oncology Group performance status; TBIL, total bilirubin; DBIL, direct bilirubin; IBIL, indirect bilirubin.

### A serum bilirubin-based nomogram predicts NSCLC patient survival

**Figure 3:**
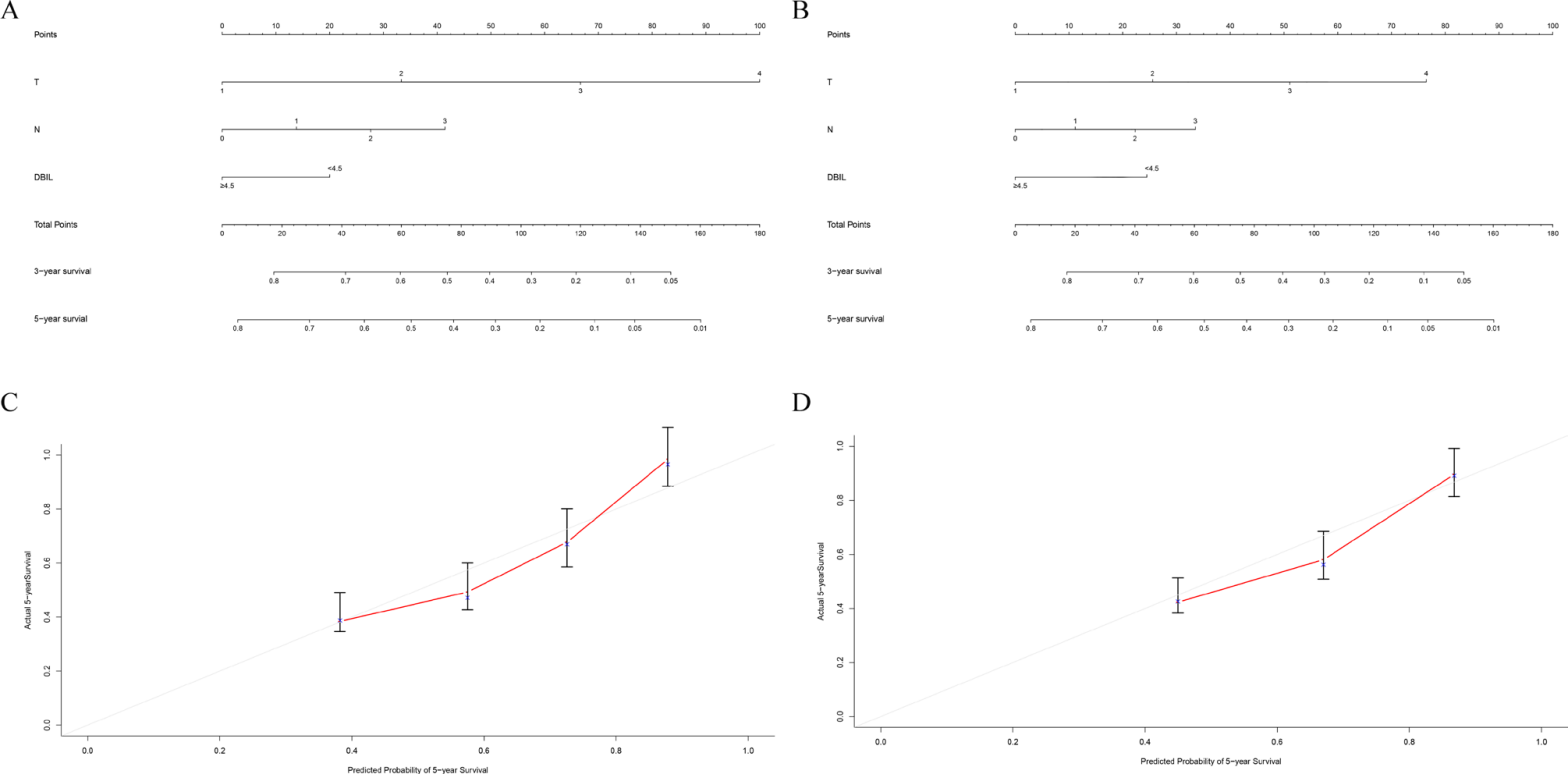
Prognostic nomogram for predicting survival in NSCLC patients The c-index values were 0.714 for the training cohort **(A)** and 0.735 for the validation cohort **(B)**. Calibration curves for 3-year and 5-year overall survival for the training cohort **(C)** and the validation cohort **(D)**.

We developed a serum bilirubin-based nomogram to predict 5-year OS rates in NSCLC patients. We included pT stage, pN stage, chemotherapy, TBIL, DBIL, and IBIL in the nomogram for patients in both the training (Figure 3A) and validation (Figure 3B) cohorts. We found that pT stage and pN stage were poor prognostic factors for survival, whereas high DBIL was a favorable prognostic factor. There results were validated by multivariate Cox regression analysis (Table 6). Calibration curves demonstrated that the predicted and actual 5-year OS rates were similar (Figure 3C and 3D).

We next compared the predictive accuracy of our prognostic model with that of the traditional TNM staging system. The Harrell’s c-indices for the nomogram were 0.714 and 0.735 compared to 0.684 and 0.701 for the TNM staging system in the training and validation cohorts, respectively. These data indicated the predictive accuracy of the DBIL-based nomogram was superior to that of the TNM staging system (*P* < 0.05).

## DISCUSSION

We found that DBIL levels are prognostic in NSCLC. Elevated pretreatment DBIL levels were correlated with longer OS in both the training and validation cohorts. Additionally, elevated DBIL levels were associated with response to first-line platinum-based combination chemotherapy in the validation cohort.

Previous studies have yielded conflicting results regarding the association between serum bilirubin levels and patient prognosis in NSCLC. We found that elevated (but within the normal range) DBIL levels were associated with favorable prognosis in NSCLC, which may be explained by the anti-inflammatory, anti-oxidative, and anti-proliferative effects of bilirubin [10]. Interestingly, Horsfall *et al.* demonstrated that higher serum bilirubin levels were associated with a lower risk of respiratory disease and all-cause mortality [8]. In contrast, Zhang *et al.* found that elevated DBIL levels were associated with poor outcomes following surgery in colorectal cancer patients [11]. Although Li *et al.* found that elevated DBIL levels were associated with longer OS in NSCLC, pretreatment DBIL level was not an independent predictor [12]. Finally, Zhang *et al.* reported that pretreatment DBIL was positively correlated with OS in NSCLC patients with *EGFR* mutations [13].

Our results indicated that reduced DBIL levels were correlated with advanced NSCLC and poor prognosis. Serum DBIL was found to be an independent prognostic indicator in both our retrospective training and prospective validation cohorts. Collectively, the data suggest that serum DBIL levels are prognostic in NSCLC.

Although TNM stage is a useful prognostic indicator, it may not be adequate for predicting survival. The predictive power could be improved by combining TNM stage with other biomarkers into a single index [14–16]. Therefore, we established a nomogram based on several pathological characteristics and evaluated whether it could accurately predict prognosis in NSCLC. We found that the prognostic model accurately predicted 5-year OS in both the training and validation cohorts. Serum DBIL level was incorporated into the model through a stepwise algorithm and the predictive power verified using calibration curves. Importantly, the nomogram was superior to the TNM staging system in predicting patient prognosis.

Our study had several strengths. First, all of the NSCLC patients were treated at a single center. Second, the analysis included both retrospective and prospective cohorts. Finally, the X-tile software was used to identify the optimal cutoff value for serum bilirubin [17]. Our study also had several limitations including the small sample size. Additionally, we only included pretreatment serum bilirubin levels in the analysis and did not test whether alterations in serum bilirubin levels throughout treatment could impact patient survival.

In summary, we have demonstrated that serum DBIL levels are prognostic in NSCLC. A DBIL-based nomogram is more accurate than the TNM staging system in predicting clinical outcomes in NSCLC.

## MATERIALS AND METHODS

### Study patients

Consecutive patients who were treated in the Department of Respiratory Medicine and Gerontology at Huai’an First People’s Hospital between January 2006 and December 2013 were enrolled in the study. The retrospective training cohort included 307 patients who underwent surgical resection between January 2006 and December 2009. The prospective validation cohort consisted of 327 patients who underwent surgical resection between January 2010 and December 2013. The diagnosis of NSCLC was confirmed histologically according to the TNM criteria (2009 AJCC criteria). All protocols were approved by the Ethics Committee of Nanjing Medical University.

Eligible patients had (1) TNM stage: I–IIIA NSCLC, (2) were not treated prior to serum collection, and (3) had complete follow-up information available. Patients were excluded if they had a history of other malignancies, hepatobiliary or hemolytic disease, and/or insufficient follow-up information available. Lab tests were performed before treatment to establish baseline bilirubin levels.

### Data collection and measurement of serum bilirubin levels

Baseline characteristics including age, sex, and smoking history were obtained through a structured questionnaire. Pathological, treatment response, and follow-up data were evaluated by physicians. Serum bilirubin levels (TBIL, DBIL, and IBIL) were measured in fasting blood samples prior to treatment using the vanadium oxidation method.

### Statistical analysis

Continuous data are presented as the mean ± standard deviation (SD) or median and interquartile range (IQR). Categorical data are expressed as percentages. The X-tile software was used to identify the optimal serum bilirubin cutoff level for sorting patients based on bilirubin levels. Differences between two groups were analyzed using χ^2^ or Mann-Whitney *U* tests. Survival analysis was performed using Kaplan-Meier curves and log-rank tests. Multivariate analysis was performed using a Cox proportional hazards model. The serum bilirubin-based nomogram was established using a stepwise algorithm and the R software. The prognostic accuracy of the nomogram was compared to that of the TNM staging system using Harrell’s c-index. *P* values < 0.05 were considered statistically significant.
